# Textile-Integrated Thermocouples for Temperature Measurement

**DOI:** 10.3390/ma13030626

**Published:** 2020-01-31

**Authors:** Waleri Root, Thomas Bechtold, Tung Pham

**Affiliations:** Research Institute for Textile Chemistry/Physics, University of Innsbruck, Hoechsterstrasse 73, 6850 Dornbirn, Austria; waleri.root@uibk.ac.at (W.R.); tung.pham@uibk.ac.at (T.P.)

**Keywords:** textiles, temperature sensor, conductivity, coatings, deposition, thermocouple

## Abstract

The integration of conductive materials in textiles is key for detecting temperature in the wearer´s environment. When integrating sensors into textiles, properties such as their flexibility, handle, and stretch must stay unaffected by the functionalization. Conductive materials are difficult to integrate into textiles, since wires are stiff, and coatings show low adhesion. This work shows that various substrates such as cotton, cellulose, polymeric, carbon, and optical fiber-based textiles are used as support materials for temperature sensors. Suitable measurement principles for use in textiles are based on resistance changes, optical interferences (fiber Bragg grating), or thermoelectric effects. This review deals with developments in the construction of temperature sensors and the production of thermocouples for use in textiles. The operating principle of thermocouples is based on temperature gradients building up between a heated and a cold junction of two conductors, which is converted to a voltage output signal. This work also summarizes integration methods for thermocouples and other temperature-sensing techniques as well as the manufacture of conductive materials in textiles. In addition, textile thermocouples are emphasized as suitable and indispensable elements in sensor concepts for smart textiles.

## 1. Introduction

Extreme exposure of the human body to high temperature can cause severe effects such as heat illness. For appropriate heat monitoring, thermometers, metallic electrodes, or sensor chips can measure human temperature directly in the doctor’s office. However, their permanent adherence to the skin, especially during outdoor activities, can lead to the wearer´s discomfort and in particular to skin irritation. This can be avoided by using textile-based temperature sensors, which can detect first signs of a heat illness outside the doctor´s office.

Heat-related illness occurs when the body stores more heat than it can release, which is accompanied by symptoms as heat stroke, heat cramps, and heat exhaustion [1,2]. Avoiding heat illness is particularly important in sports and the mining industry [2,3], which can be achieved by monitoring the temperature of the human body by textile sensors [4,5,6,7,8]. 

Integrated sensors should exhibit significant flexibility and low weight characteristics [9], which is important for health care applications such as body temperature measurement [10]. In health care, the body temperature is detected on the human skin [9]. This data can be used to investigate wound-healing processes, assessing patient comfort, or monitoring temperature development during sleep [9]. The integration of thermocouples into textile structures is a straightforward solution for temperature monitoring [9].

A thermocouple consists of two different conductive materials, which are connected at one point/form a closed circuit. One connection point is termed the measuring junction and the second can be regarded as a reference junction [11]. The thermocouple develops a voltage between two different materials of wires that can be used to measure temperature. Due to temperature changes, a voltage is generated between the different materials. Therefore, output voltage is related to the change in temperature [12]. This effect was first described by Seebeck in 1826. He discovered that a current flowed in a closed circuit between two dissimilar wires when two junctions are exposed to different temperatures [11,13,14]. The output voltage (*∆U*) is calculated by Equation (1):

*∆U = α × ∆T* (1)

where *α* is the difference in the Seebeck coefficient of the two metal conductors, and *∆T* is the temperature difference between the cold and hot junction [12].

**Figure 1:**
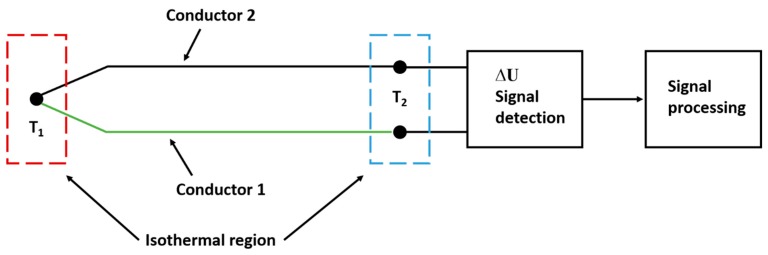
The model of thermocouple detection and signal processing according to [13].

In 1834, Peltier observed a current flow when a junction of two different wires was cooled or heated [11,13,14]. Twenty years later, in 1854, Lord Kelvin (W. Thomson) concluded that the current flow results from a temperature gradient in the conductor [11,13,14]. Figure 1 shows the model of a thermocouple, which uses two different conductors, a signal detection, and signal processing unit. The dotted boxes around the measuring and reference junctions show that these regions are isothermal. Isothermal regions do not contribute to the voltage detection (*∆U*). The blue and red marked regions are set to different temperatures [13]. 

**Figure 2:**
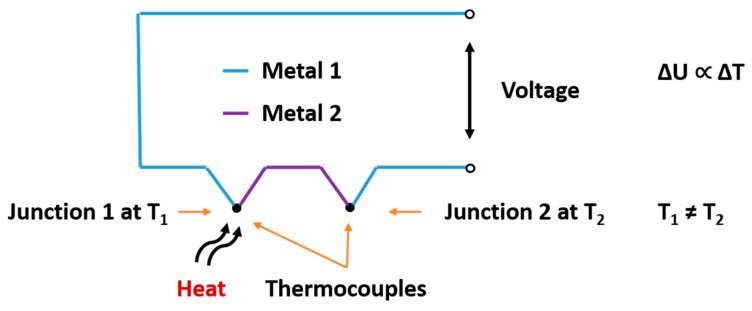
The first junction is heated to T_1_ while the second junction stays at temperature T_2_. According to [15], this results in an analog voltage signal.

Based on this model, a thermocouple pair can be constructed. A thermocouple pair generates a voltage when two junctions are set at different temperatures (Figure 2). The change of temperature at one junction leads to a voltage change across the thermocouple pair, which is proportional to the change in temperature. 

Temperatures have been measured using various thermocouple assemblies such as thermocouple tips, [16], multipoint thermocouples [17], and a combination of dissimilar metal wires (platinum–rhodium alloy) [18]. Thermocouples can also be formed by printing techniques using iron, nickel, and copper inks [19]. The latest reports show that thermocouples can also be formed using two conductors of the same material with different material thicknesses [20,21,22].

Recent literature reports the invention of single-metal thermocouples consisting of different conductor widths, which are used for temperature detection [20,21,22].

**Figure 3:**
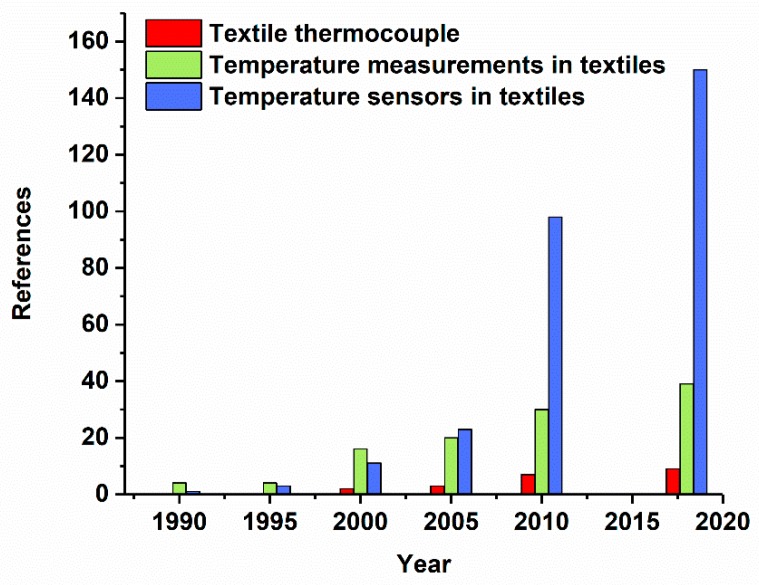
Number of concepts for the integration of temperature measurement systems and thermocouples into textiles published until 2019. The SciFinder database was used for the literature search with the key words textile thermocouples, temperature measurements in textiles, and temperature sensors in textiles.

Over the last three decades, the interest in the integration of temperature measurement systems into textiles has significantly grown. In Figure 3, this evolution is shown by the number of concepts for “Textile thermocouple”, “Temperature measurements in textiles”, and “Temperature sensors in textiles” published in the years 1990, 1995, 2000, 2005, 2010, and 2019.

Regarding the concept of textile thermocouples, the slight increase in publications shows that there is no precise definition of textiles thermocouples in literature. In general, thermocouples can be integrated into textiles by various techniques:

- Screen printing of conductive polymers [23]
- Sputter deposition of metal stripes [24]
- Soldering of metal wires [25]
- Using electro-conductive glues [26]
- Interweaving of metal wires [27]

For the ease of the reader finding the summary of five techniques, we have summarized them in Table 1, which permit having a quick overview of the methods.

Ziegler and Frydrysiak defined that textile thermocouples may be manufactured from thermoelectrodes consisting of functionalized textiles of woven, non-woven and knitted fabric threads, twisted multifilaments, yarns, and fibers. The functionalization of textiles can be conducted with conductive nanoparticles or electro-conductive polymers [26]. A general definition of textile thermocouples used in this review could be stated as:

- Textile thermocouples detect changes in temperature and consist of an indispensable conductive textile matrix with a textile character.

Textile thermocouples should combine the flexibility and light weight of textiles with the conductive property of the conductor material, which can be defined as a truly textile thermocouple. 

In this review, various integration methods and conductor materials for the construction of thermocouples in textiles will be described. This review discusses how textiles serve as appropriate carrier materials for the integration of temperature sensors. Different aspects of manufacturing conductive textiles will be shown. An outlook is given, which emphasizes the advantages and limitations of thermocouples in textiles.

## 2. Concepts of Thermocouple Construction in Textiles

Different thermocouples have been used to measure temperature on woven, non-woven, and knitted textiles. 

**Figure 4:**
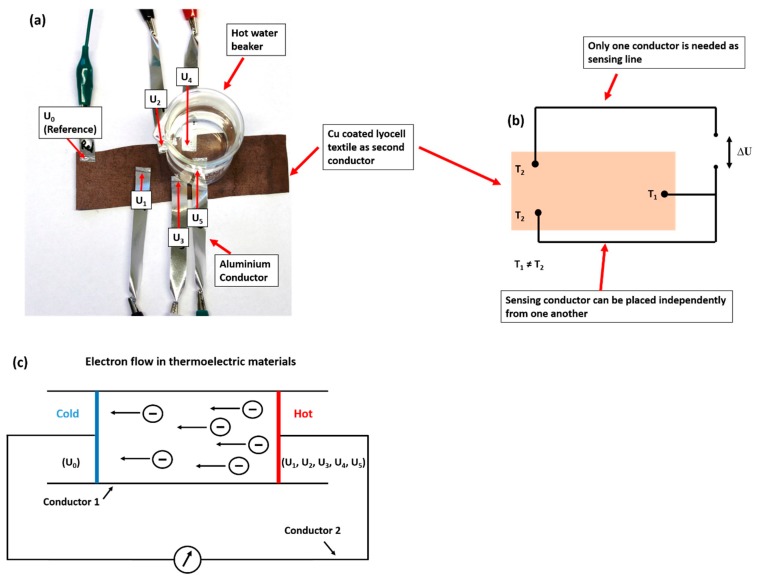
The construction of five thermocouple pairs (**a**), the description of electron flow in thermoelectric materials (**b**) according to [12], and (**c**) an electrical circuit. U_0_ is the reference junction and U_1_, U_2_, U_3_, U_4_, and U_5_ are measuring junctions.

Figure 4a shows the construction of five thermocouple pairs, which consist of five aluminum conductor strips and a large copper-coated cellulose fabric as a second conductor. Using the copper-coated cellulose textile as a conductor material makes the thermocouple construction more flexible compared to metal wires. The size of the copper-coated cellulose textile can be varied, which allows the positioning of additional thermocouples independently (Figure 4b). This thermocouple construction needs only one conductor as a sensing line. Figure 4c shows a scheme of electron flow in thermoelectric materials. It describes the formation of a temperature difference across a conductor when two junctions (regions) are set to different temperatures. The hot junction (region) generates more free electrons compared to the cold junction. Thus, an electron flow occurs from the hot to the cold junction (region) [12]. 

Thermocouples were manufactured from conductive poly(3,4-ethylenedioxythiophene), poly(4styrenesulfonate) (PEDOT-PSS) and polyaniline by screen printing on woven cotton textiles [23]. In addition, thermocouples were used to detect resistivity and temperature as a function of time (up to 35 h). Thermocouple assemblies made from PEDOT-PSS and polyaniline showed a Seebeck coefficient of 18 µV/K comparing to 15 µV/K copper polymer assemblies [23]. In a further composition, thermocouples were manufactured from several textiles such as polyacrylonitrile staple fibers, steel staple fibers, a silver-coated polyamide thread, a knitted steel fabric, a woven polyacrylonitrile fabric, and a graphite non-woven textile [26]. The electrical signal generated from thermocouples was used to measure the temperature in the range of 30 to 120 °C [26]. The thermocouple was constructed from L-shaped copper and constantan (Cu/Ni) stripes on polypropylene textile, which were formed by magnetron sputter deposition. Comparison with a commercial thermocouple indicated no difference in temperature detection [24]. Temperature sensors were constructed from copper–nickel wire thermocouples, which were soldered onto a firefighter´s glove [25]. 

Thermocouple sensors have been manufactured from wires to monitor the thermal situation in socks and gloves [9]. The body heat regulation was monitored by a sensor-based platinum array outside of the garment [9]. Thermocouple sensors were manufactured from copper and constantan wires and were used to detect temperature at 12 different locations in T-shirts [27]. Consequently, a temperature distribution depending on the garment´s size and a distance from the body could be measured [27]. 

**Figure 5:**
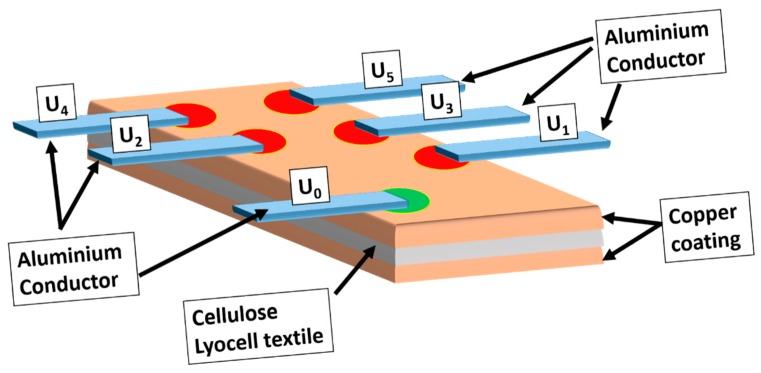
Copper-coated cellulose textiles used as a conductor matrix for temperature measurement.

Copper-coated textiles can be used as flexible and lightweight conductor materials in a thermocouple array (Figure 5). The number of conductive lines can be reduced to measuring junctions (red spots), and a reference junction (green spot) can be formed by the attachment of five aluminum conductors (U_0_, U_1_, U_2_, U_3_, U_4_, and U_5_).

Thermocouples were used to measure heat flux through polyester and polyester/cotton fabric with different weaves (plain, satin, and twill [28]. The fabric´s temperature was detected at thermocouple points, which were related to reference points at room temperature [29]. Table 1 discloses various implementations of thermocouples in textiles and their influence on the flexibility of the entire structure, which became stiffer, especially by gluing and soldering.

The thermocouples shown in Table 1 in this summary were made from metal wires, fibers, and yarns, which increase the textile´s weight and reduce its flexibility. The incorporation techniques mentioned often (Table 1) integrate thermocouples in textiles, thus resulting in discomfort and increased stiffness. Further scientific work is needed to deal with these inconveniences and to manufacture a truly textile thermocouple. 

## 3. Other Strategies for Temperature Measurement in Textiles

There are several strategies to measure temperature in textiles such as Positive Temperature Coefficient (PTC), Negative Temperature Coefficient (NTC), Resistance Temperature Detector (RTD), and fiber Bragg grating (FGB). These strategies determine the shape of temperature sensors, which are manufactured by weaving, lithography, adsorption, screen printing, embroidery, knitting, and gluing, using conductive carbon paints and chemical vapor deposition (CVD).

Temperature measurements were conducted with a PTC resistive temperature sensor. It was manufactured as a thin film capacitor with gas/humidity sensitive polymers on a 50 µm Kapton substrate, on which two electrode lines were formed by lithography. The temperature sensor was woven into a textile (width = 45 mm, length = 200 mm) [30]. PTC sensors also were manufactured from an activated carbon fiber cloth by an electrothermal swing adsorption method [31]. A PTC sensitive polyamide foil (KAPTON) was manufactured by screen printing carbon polymer composites, while polyethylene and rubber were used as binder materials. The PTC-sensitive foil-detected temperature increase from 30 to 42 °C as a function of resistance [32]. PTC sensors were used to investigate the heating properties of 40 µm embroidered flexible polyurethane-coated copper filaments. PTC sensors on cotton fabrics recorded the increase in temperature as a function of resistance on embroidered PU-Cu composites. [33].

The temperature in textiles was detected by NTC sensors, which were manufactured from thermosensitive polyvinylidene fluoride (PVDF) fibers of 2 to 6 cm in length and 0.15 mm in diameter. Their active sensor area was formed by thermosensitive, polymer conductive pastes, which were manufactured from multiwall carbon nanotubes (MWCNT) and poly(methylmethacrylate) (PMMA) [34]. In another report, the sensitivity of conductive fabrics to different temperatures was investigated by cotton and silver yarns. The sensitivity was related to the fabric’s resistance, which was measured between two brass blocks (500 g each) at a pressing force of 25 N in an oven [35]. Five sensing yarns were incorporated into 2 mm diameter channels on a knitted sock and measured temperature on the skin. One temperature-sensing yarn consisted of a copper wire, six polyester yarns, one NTC thermistor, and a polymer resin, which were processed by a flatbed knitting machine [36]. 

In a further method, the temperature in textiles was measured by RTD sensors consisting of Kapton and Ti/Au conductors. The sensors were produced in a commercial band weaving process. The sensors were glued with conductive epoxy to metal strips, which were connected to a measurement device [37]. Another RTD detector was manufactured from nylon-6 after electrospinning and functionalized with multiwalled carbon nanotubes (MWCNTs) and polypyrrole (PPy). The samples were treated in the pyrrole vapor for 48 h and connected to two copper electrodes with conductive carbon paint [38]. RTDs were also made from a 100 nm platinum-coated plastic strips of 67.5 mm length and 500 µm width. The strips were woven in Kapton textiles at a distance of 200 µm and were used for temperature measurements [39]. In a further construction, RTD sensors were manufactured from nickel, copper, and tungsten wires on a temperature sensor fabric. These wires were knitted in the middle of a polyester fabric using a flatbed knitting machine. In the design, electrical short circuits were avoided, and a resistance of 3 to 130 ohms was determined during temperature measurements [40]. Flexible temperature and humidity sensors were made from graphene woven fabrics (GWF) on flexible polydimethylsiloxane (PDMS) films, which were deposited by CVD. The change in resistance, which was recorded as a function of temperature from 20 to 60 °C, demonstrated the use as a sensor [41].

A further method of temperature detection in textiles is the use of reflected wavelengths, which was caused by angular deformation. This deformation was used during temperature measurement in fiber optical sensors, which are based on the fiber Bragg grating (FBG) method and are woven in socks. The FBG sensors are made of silica core and plastic substrates, which provide the material a durability and light weight [42]. As an example, the body temperature can be detected by FBG sensors, which are woven into fabrics and embedded into a polyester resin [10]. The FBG method was used to investigate the temperature from 30 to 70 °C of liquid mixtures (water/glycerin) with a negative thermo-optic coefficient of −5 × 10^−4^ °C^−1^. The FBG fiber was placed in an aqueous solution with the mixture solution. The Bragg wavelength of the FBG fiber was measured while heating the solution [43]. The structural state of textiles can be monitored when the beam IR laser (1064 nm) impinges on the surface, leading to a thermal gradient of 100 °C. The FBG temperature sensors measured radiation on the polymer surface, which can be used for flame or energy attack detection [44]. In addition to the FBG method, plastic optical fibers (POFs) were used as temperature sensors. During that method, the temperature was measured as a function of the intensity, which was caused by the thermal bending of the fiber. The POF sensors consisted of polymethyl methacrylate and fluorinated polymers, which were used as core and cladding [45].

The POF was used in chirped fiber Bragg grating sensors of 10 mm length, which indicated a sensitivity of −191.4 pm/°C. These sensors measured temperatures along the grating length, which were designed for biomedical treatments and thermotherapies [46]. For biomedical application, POF was used due to the rapid production of POF grating devices, which worked below 248 nm and 266 nm UV wavelengths. This led to the manufacture of chirped POF-FBG sensors with a higher sensitivity and better biocompatibility compared to silica-based sensors [47]. In biomechanical investigations, multiple FBG sensors showed a sensitivity of 10.6 pm/°C, which were connected in serial on textiles. This connection formed a temperature sensor network with multiple points, with which temperature values from 20 to 130 °C were measured [48].

Different techniques and materials are summarized next, which permit temperature measurements in textiles (Table 2).

## 4. Aspects of Manufacturing

### 4.1. Integration of Electrically Conductive Elements

**Figure 6:**
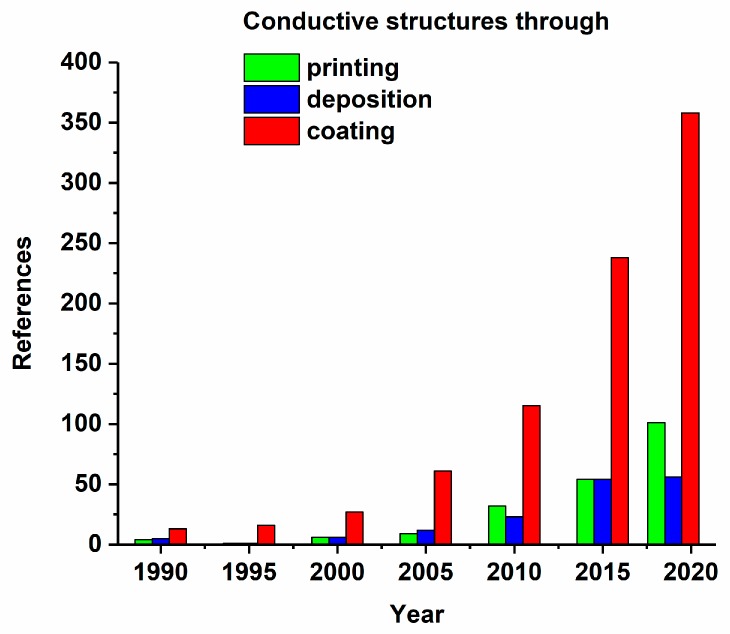
Research activity in coating, deposition, and printing processes to manufacture conductive structure in textiles.

The central element to integrate sensors in textiles is to achieve stable connections. Figure 6 shows an output of a literature search on manufacturing techniques in textiles. The concepts are highlighted with green for “conductive printing on textiles”, blue for “conductive deposition on textiles”, and red for “conductive coating on textiles”. The research dynamics of the three approaches increased over the period of 29 years, which indicates the growing interest in techniques to miniaturize textile sensors. 

In general, the coating or deposition of conductive materials has more advantages than the soldering, welding, and weaving of metallic wires or sensors in textiles. The classification of products was related to the aspect by which the coating or deposition of materials can be conducted on large textile areas and spatially defined structures such as yarns or fibers. The coating or deposition of conductive materials leads to a conductive thin layer formation on substrates compared to soldering. Thus, coated conductive textiles are flexible and have a higher motion of freedom compared to soldered textiles. The scientist makes the choice of textile materials because it is more likely that different materials and textile structures will be used in each scientific field. The properties of conductive materials are determined by the firm or loose textile structures, their swelling properties, and the amount of reactive groups.

The advantages of coated or deposited materials are the low weight and the thin coating thickness. Thus, conductive coatings better retain the flexibility, bending, and stretch properties of textiles compared to rigid metallic wires or sensors. Consequently, the combination of conductive coatings and deposits on textiles also contributes to the construction of truly miniaturized textile thermocouples. A truly textile thermocouple was described in the Introduction. 

The manufacturing of electrically conductive substrates on textiles can be performed by different techniques e.g., soldering, stapling, and bonding components through conductive adhesives. Soldered substrates do not withstand the bending of textiles. Stapled substrates increase the wear and tear of textiles due to the rigid structure and reduce the freedom of movement. The connection between textiles and conductive substrates can be made by a flexible conductive material [49]. 

Representative examples for such materials are bicomponent fibers that include poly(vinylidene fluoride) as the sheath material, carbon black, and high-density polyethylene as the core material. Bicomponent yarns were made during the melt spinning process with two screw extruders consisting of the core and sheath material [50]. Conductive core–sheath yarns of copper core filament and cotton sheath were manufactured through the Dref-3 friction spinning method. The core–sheath yarns were made of copper filaments as a core and cotton fibers as sheath. These yarns showed a resistance of 3 to 28 MΩ and a shielding effect of 760 to 860 MHz at a core sheath ratio of Cu 0.26 gram per meter and cotton 0.13 grams per meter [51]. Elastic conducting inks were made of Ag flakes, fluorine rubber, and fluorine surfactant, which showed a conductivity of 182 Scm^−1^ during stretching. These materials were used as wearable electromyogram sensors to detect the signal activity of the muscles of the forearm. Elastic conductor inks were printed on polyimide stencil masks, which formed flexible conductor wires on the upper side of the textile and an elastic conductor vital electrode on the lower side of the textile [52]. Wearable electronic textiles were created by a lockstitching method and were used in apparel textiles. Conductive assemblies were made by stitching conductive threads (such as silver, aluminum, stainless steel, copper, and carbon) on the surface of a cellulose and stitching thermofusible threads (polyamide, polyolefin, and polyvinyl) on the polyester/elastane [53]. 

The surface of cotton textiles was rendered conductive by impregnating with carboxylated multiwall carbon nanotubes by dispersion. The cotton fabrics were treated with aqueous NaOH/urea mixture at −10 °C for 1 h and showed a low electrical resistivity of 281 Ω cm [54]. The formation of conductive textiles was manufactured through screen printing of the FeCl_3_ and by applying high voltage from 5 to 30 kV during the coating of pyrrole by vapor deposition. The high voltage along the polypropylene-coated fabric stabilized pyrrole monomers during vapor deposition [55]. Non-conductive epoxy surfaces were laminated with copper sheets by the pressing method. Afterwards, these materials were activated with stannous/palladium chloride particles. The epoxy substrates were made conductive after 20 h of electroless copper plating [56]. The formation of conductive tracks of 1.5 and 4.0 mm was achieved on cotton textiles by the reduction of silver nitrate from sodium borohydride during the spray deposition. Subsequently, the silver seeded tracks were plated selectively with copper during the electroless process from aqueous solution [57]. Copper foils were used to form circuits in cloths, which consisted of silk organza fibers. The electrical circuits structure was manufactured by embroidery and by an industrial sewing machine [58].

Additionally, conductive coatings on cotton fabrics were manufactured by the surface activation in NaOH and poly(diallyldimethylammoniumchloride) solution. The activated cotton fabrics were impregnated with NaBH_4_ in aqueous solution, and afterwards, a silver nitrate solution was added to the fabric. The cotton fabrics were completely coated with silver nanoparticles after the reduction of silver ions by NaBH_4_ [59]. 

Coated textiles powered small consumers without the use of metal wires and impart electromagnetic shielding properties by the examples below. Conductive woven cellulose fabrics power a light-emitting diode (LED) at 20 mA. The copper layer was formed after silver seeding through an electroless deposition in alkaline solution comprising a Cu L-tartrate complex and formaldehyde [60]. 

**Figure 7:**
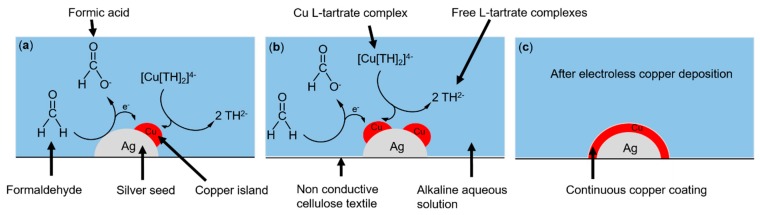
The electroless copper deposition method conducted on cellulose textiles, where the tartrate complex (TH) is a free L-tartrate ligand (**a**). The copper deposition continues on Ag seed (**b**), which leads to the copper layer formation (**c**).

Figure 7 describes the electroless deposition method of copper on silver seeded cellulose textiles in alkaline solution. The silver seeded textile is dipped into copper sulfate, formaldehyde, and potassium hydrogen L-tartrate solution (Figure 7a). Formaldehyde is a chemical reducing agent, which reduced copper ions on silver seeds from the copper tartrate complex (TH) to metallic copper (Figure 7a,b). When the deposition proceeds (Figure 7c), copper islands are formed on silver seeds, which then grow to a continuous coating [61].

Cotton fabrics imparted conductive properties after the in situ deposition of copper particles and repeated dipping steps in the CuSO_4_ and Na_2_S_2_O_4_. Copper-coated textiles can be used as flexible and light materials. The copper-coated cotton fabrics showed a shielding property of 6 dB, 10 dB, and 13 dB when the fabric was dipped in the copper sulfate solution 50, 100, and 150 times, respectively [62].

A low electrical resistance of textile material can be achieved also by treatment with conductive polymers after impregnating, vapor deposition, and melt mixing methods. Electro-conductive fabrics can be made from wool, cotton, and silver-coated acrylic yarns. Textiles composed of silver-coated wool yarns and silver-coated cotton/acrylic were used as heating elements in textiles [63]. The incorporation of conductive material during fiber formation also leads to polymer fibers with conductive properties. 

A non-woven poly(ethylene oxide) (PEO) matrix was mixed with 3 wt % multiwalled carbon nanotubes (MWNT), which formed conductive polymer composites by an electrospinning process. The maximum electrical resistance of PEO/MWNT composites changed when exposed to methanol, dioxan, and toluene vapors [64]. Conductive monofilaments composites were formed from carbon nanotubes (CNT), polypropylene, poly(ε-caprolactone) (PCL), and polypropylen substrates. The materials manufactured from 50%PP/50%PCL/4%CNT composites showed a resistivity of 1.1 Ωm at 154 °C [65].

Coated textiles were used for temperature detection in the range of 15 to 57 °C. Conductive polyamide fabrics of 17% Lycra and 83% Tactel (5 cm × 1 cm) were coated from aqueous solution with poly(3,4-ethylenedioxythiophene)-poly(4-styrenesulfonate) (PEDOT-PSS). PEDOT-PSS-coated fibers were exposed to environmental temperatures of 15 and 45 °C. The electrical resistance of coated fibers decreased with increasing temperature [66]. Conductive polyester yarns were manufactured from copper nanowires and a silicon rubber substrate during a dip-coating. The coated polyester yarns were used as stretchable heating fibers. The composites were woven into a heating fabric and connected to a microcontroller unit to manufacture wearable and smart personal heating systems [67].

### 4.2. Aspects of Aging

The information about the working property of textile sensors, conductive materials, and protective clothing over their entire lifetime still remains underreported in the literature. For firefighters’ protective garments, the aging of materials under environmental conditions reduced due to the low shear resistance even after a short period of time. The mechanical strength of textiles was reduced by up to 80% before a damage was detected visually [68]. The thermal degradation in aramid/basophil firefighter cloth occurred before the optical change was detected. After a convective heat of 80 kW/m^2^ and a radiant heat exposure of 40 kW/m^2^, the mechanical properties of fabrics in a tensile test decreased by 40% and 60%, respectively (660 N) [69]. 

The advantages of thin flexible and electric coatings are the good conductivity and the low impact on textile properties such as their handle, flexibility, and density. Possible problems with coatings are their corrosion and insufficient adhesion between the textile and the coating substrate [70]. Polyester fabrics were coated with polypyrrole during incubation in saline substrate for up to two weeks at 37 °C. They exhibited a resistance in the range of 10^3^ to 10^4^ Ω/square. It was observed that the decrease in electrical conductivity was related to the oxygen uptake during incubation and due to cracking of the coating [71]. 

**Figure 8:**
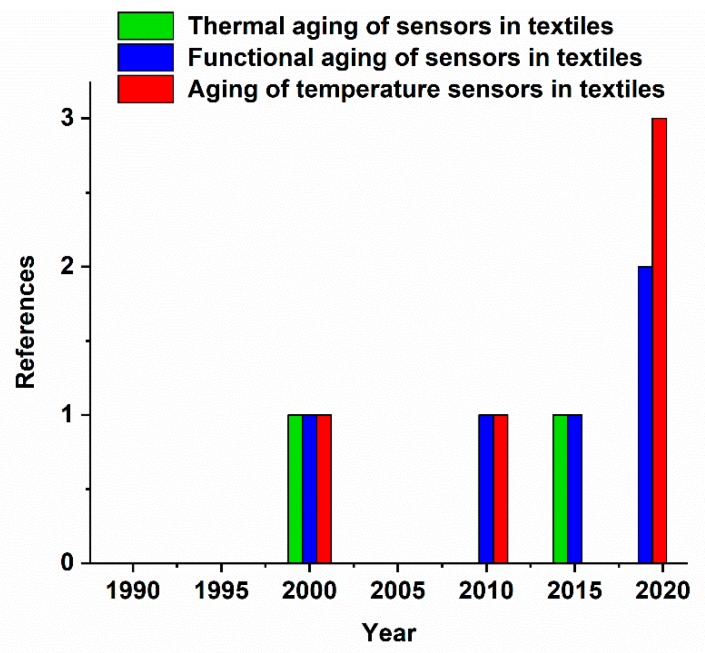
The effects of aging on sensors in textiles.

Figure 8 shows the results of a literature search on sensor aging containing three different search concepts. The concepts are highlighted with green for “thermal aging of sensors in textiles”, blue for “functional aging of sensors in textiles” and red for “aging of temperature sensors in textiles”. There are gaps in the literature dynamics of all concepts in the last 29 years, which do not provide a general concept for sensor aging in textiles. During the period of 2000–2019, the total number of references for the concepts of thermal aging of sensors in textiles, functional aging of sensors in textiles, and aging of temperature sensors in textiles were two, five, and four respectively, which indicates low scientific interest in degradation and aging in temperature sensors in textiles. 

### 4.3. Aspects of Life Cycle of Conductive Textiles and their Regulation

Electrically conductive textiles will gain more importance for mass consumer applications. Thus, a new kind of waste will be formed. The market of smart textiles and wearable electronics is estimated to grow from $20 billion in 2015 to $70 billion in 2025 [72], which emphasizes their importance for the mass consumer application. According to the European Commission in 2017, the high potential of wearables on the European market was reported in the orientation paper about smart wearables [73]. 

The waste difficulties of e-textiles can be overcome by implementing an appropriate eco-design strategy, which include e-textile labeling and the use of compatibility standards [74]. The impact of new waste could cause toxicological stress on human health, the ecosystem, resources, land use, and water use. These negative impacts can be reduced through the life cycle assessment at an early stage of the development, which assesses the potential environmental impact of products and identifies solutions for preventing pollution and decreasing the resource consumption [75].

As an example for a toxicological assessment of a surface of a modified textile, the coating of polyester and cotton fabrics with nano-metal oxides such as CuO and ZnO was studied. Fabrics treated with water and ethanol showed a release of CuO and ZnO nanoparticles up to subtoxic concentrations of 1 µg/mL in A549 cells. At a low concentration up to 10 µg/mL, there was no acute toxicity observed in lung epithelial and macrophage cells compared to an exposure of 100 µg/Ml [76]. 

Besides the toxicological evaluation, the production of e-textiles in industrial processes has to comply with the legal requirements of European Eco-design, which describe the development of energy-related goods. Future goods design and sustainable material management can be related to the U.S. Environmental Protection Agency, which regulates the life cycle of products during their manufacture [77]. The use of metals for conductive substrates in textiles should be regarded as a metal finishing process, which is conducted by the industry. Consequently, the industry is bound by the laws of regulation for metal finishing such as the Resource Conservation and Recovery Act, the Clean Air Act, and the Clean Water Act (CWA). The CWA includes the Effluent Guidelines and Standards for Metal Finishing and the Effluent Guidelines and Standards for Electroplating. These guidelines and standards are mandatory for facilities dealing with electroplating, coating techniques, electroless plating, printed circuit board production, chemical etching, and milling. The standards determine the concentration of pollutants in wastewater from the above-mentioned processes, which are described in milligrams per m^3^ [78].

## 5. Temperature Sensors and E-Textiles

### 5.1. Wearable Heaters

Wearable heaters also record temperature profiles as a function of time and can be used in many applications e.g., thermotherapy. In many cases, a combination of heating device and temperature sensor is implement with the aim to control heat generation and to avoid over temperature.

Wearable heaters, which are manufactured from Ag nanofibers (AgNF) on polyethylenterephthalat (PET) and polyimide (PI) by electrospinning, can be affixed to the skin. Heaters were connected at both ends by Cu wires, while the current was applied from the power supply for heat generation. The AgNW (nanowire) heater on the PI substrate shows a considerably stable temperature of 42 °C during a stretching test up to 90%. The use of SiO_2_ as a passivation layer on AgNW heaters can retard Ag oxidation and allow the detection of temperature up to 250 °C [79]. 

Wearable and stretchable heaters were made from PEDOT:PSS, polyurethane, and reduced graphene oxide films, which can be applied in thermotherapy. They imparted an electrical conductivity of 18.2 Scm^−1^ and withstood elongation up to 530%. The temperature distribution of composite films was measured in the middle when voltage was applied by two copper wires [80]. Heaters were also manufactured from Ag NWs (nanowires), PEDOT:PSS, and PET materials, which withstood a temperature of 120 °C [81]. Stretchable heaters were also fabricated from graphene fiber (GF). The GFs were embroidered into cotton fabric and withstood finger bending and wrist movement. The temperature was recorded by an infrared camera [82].

Flexible and stretchable heaters were manufactured from carbon nanotubes (CNT), copper foil, and silicon elastomers [83]. Flexible and stretchable heaters were constructed from copper-coated polyacrylonitrile fibers, which can operate at temperature up to 328 °C. These heaters were manufactured from copper-coated fibers by electroplating on glass substrates [84]. Flexible heaters were manufactured from nylon-coated fabric, which was coated with Ag NWs and rubber shape memory polymer during dip-dry and spray coating. Bending, rolling, gripping, and rubbing did not show any damage of the heaters [85].

Stretchable and conductive heaters were manufactured from poly(3,4-ethylenedioxythiophene):poly(4-styrenesulfonate) (PEDOT:PSS) and sodium dodecyl sulfate on cotton and polyurethane fabrics by dip coating. The temperature changes were investigated with a digital thermometer while IR images were recorded with an infrared camera [86]. Stretchable heaters were used in thermotherapy, which were produced from styrene–butadiene–styrene and Ag NW substrates. These substrates formed a mesh by thermal welding and heat treatment [87].

In thermotherapy, stretchable heaters could increase the blood flow near the wrist. The heaters were manufactured from kirigami–aluminum paper, thin elastomers of silicon polymer, and polyethylene terephthalate films, and these could be stretched to 400% at a temperature of 40 °C [88]. Stretchable heaters were also manufactured from copper wire/alumina/polyimide composites. These composites showed a high visible light transmittance up to 91.4% and reached temperatures up to 300 °C. They withstood 100 stretching and relaxation cycles at 30% strain [89]. Stretchable and wearable heaters were manufactured from CuZr and poly(dimethylsiloxane) (PDMS), which could be used at 70% elongation. They were used as portable patch units on human hands and reached temperatures up to 50 °C [90]. Stretchable heaters produced from Ag nanowires and polydimethylsiloxane (PDMS) substrates were used to heat human skin. A constant temperature of 50 °C could be observed up to 40% strain [91].

Temperature measurements were conducted by conductive substrates in textiles, which formed sensors and flexible electronic structures. Flexible electronic circuits were made by coating 35 nm Cr substrates by photolithography and 25 nm Al_2_O_3_ substrates by atomic layer deposition on Kapton E materials. Electronic circuits were integrated through the commercial weaving process integrated in textiles. They formed woven temperature sensors, which operated in the range of 20 to 100 °C [92]. Flexible and conductive polyester fabrics were manufactured from ploly(3,4-ethylenedioxythiophene):poly(4-styrenesulfonate) (PEDOT:PSS), 15 wt % graphite, and dimethyl sulfoxide mixtures by coating. These fabrics were used as thermoelectric (TE) textiles, which measured temperatures up to 398 K and showed a power of 0.025 µWm^−1^K^−2^ [93].

Bimodal sensors were used to detect temperature and pressure simultaneously by making use of a piezo-thermoresistive organic conductor and a dialectic substrate. The dielectric substrate was composed of poly(vinylidenefluoride-trifluoro-ethylene) and BaTiO_3_ nanoparticles. When the human finger pressed on the bimodal sensor, a pressure of up to 0.03 N/mm^2^ and a temperature of up to 35 °C were measured [94]. 

### 5.2. Sensor Integration in Textiles

**Figure 9:**
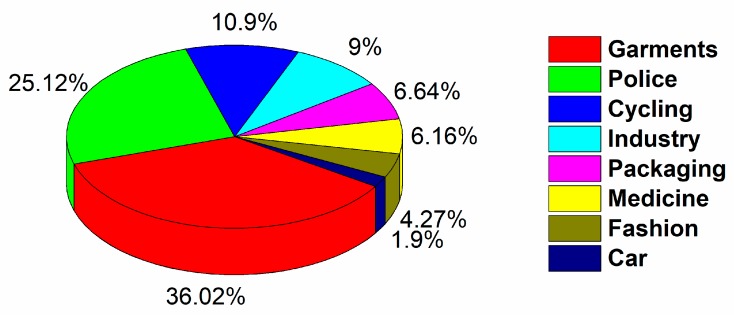
Eight areas for sensor integration in textiles in November 2019.

Figure 9 shows eight possible application areas, where the integration of sensors in textiles is of interest. The temperature detection already has been investigated in functional garments, sport garments, the automobile industry, medical institutions, security packaging, and the fashion industry. The future seamless compatibility of sensors with textiles will increase their wearing comfort and lead to prototypes, which can be produced on an industrial scale. 

### 5.3. Body Sweat/Moisture and Heat Transfer in Textiles

Besides sweat, water content influences the wearer´s comfort in textiles. The presence of water in textiles increased the mass and reduced the heat transfer in sport and protective clothing [95]. Textiles with high water vapor permeability can transfer moisture from the skin through the textile into the environment, which continuously keeps the human body in thermal equilibrium.

Therefore, the transmission of water vapor was recorded as a function of air temperature and relative humidity in polytetrafluoroethyle (PTFE) laminated with nylon fabric, woven cotton fabric, polyester fabric (laminated with polyurethane), and hybrid PTFE membranes. The transmission of water vapor was high at high air temperature and low relative humidity [96]. 

In addition to the body motion, health condition can be monitored by using biocompatible and stretchable carbon nanotube-based electrodes (CNTs), which are used to detect sweat [97]. Sweat also can be detected by a wearable colorimetric pH sensor, which provides information on the metabolic state and activity of a patient. The collection of sweat in T-shirts was investigated on textile biosensors in health management [98]. 

**Figure 10:**
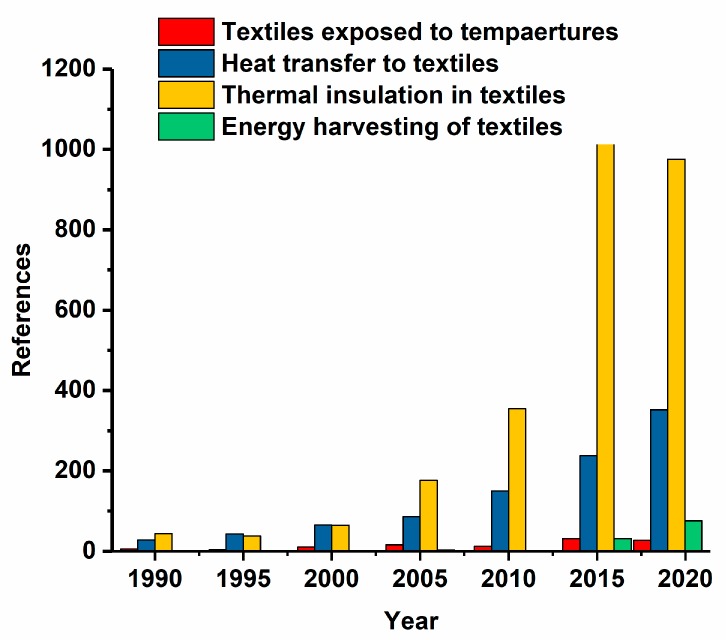
Temperature measurement in textiles and their use for energy generation.

Figure 10 shows the increase in the literature on concepts, which are related to thermal effects and energy generation. The concepts are highlighted with green for “thermal insulation in textiles”, yellow for “heat transfer in textiles”, blue for “textiles exposed to temperature”, and red for “energy harvesting in textiles”. Energy harvesting in textiles is a new fast growing field. Its role will be significant with the development of miniaturized temperature sensors that seamlessly adapt to textiles.

The thermoelectric effect also can be used to generate electrical energy from temperature differences between a human body and the environment. 

As an example, the heat of the human body was used to power a flexible thermoelectric glass fabric, which was formed from eight thermocouples consisting of Bi_2_Te_3_ and Sb_2_Te_3_ films. It indicated an output voltage of 28 mWg^−1^ (ΔT = 50 K) [99]. The temperature of the human body was detected by polyethylene (PE) and polyethylene oxide (PEO) substrates, which were melt mixed with 40 wt % Ni microparticles. The PEO/PE matrix treated with 40 wt % Ni showed sensitivity as temperature sensors of 0.3 V/°C in the range of 35 to 42 °C compared to 50 wt % [100]. The skin temperature was measured by an embedded wire sensor, which was composed of aluminum carbon epoxy composites. These composites detected a higher skin temperature compared to multiple thermistors [101]. 

## 6. Outlook and Future Perspectives

The coating of textiles with metals is a key technology for the miniaturization of low weight textile thermocouples. The metal coating follows the structure of the textile and covers its surface with a thin conductive metallic layer. The advantages of thin conductive coatings are the ability to form different geometries on small surfaces and provide a better flexibility compared to thicker substrates. Combining the conductivity of metal coatings with a fabric’s flexibility, light weight, and stretch can provide substantial progress in miniaturized textile thermocouple construction. The combination of textiles with low weight thermocouples will improve the sustainability of the assembly. 

Using a thermocouple is a simple way to measure temperature in textiles. Conductive thin-coated textiles can be used for a thermocouple construction, which measures temperature based on an electrical signal. There is a growing demand for miniaturized temperature measuring methods in textiles in the near future. 

Besides the functionality of a device, material costs will also determine the selection of conductive parts. The use of silver as a conducive material for the manufacture of wearable heaters can be explained by its high electrical conductivity of 6.3 × 10^7^ Sm^−1^ compared to that of copper, which is 5.9 × 10^7^ Sm^−1^. Despite the lower cost of copper ($6.7/kg) compared to silver ($510/kg), immediate oxide layer formation on the copper surface makes its application difficult. Conductive PEDOT/PSS substrates (2 × 10^4^ Sm^−1^) are very expensive ($167,000/kg) and may not be suitable for the large-scale production of flexible substrates [102]. 

The durability of conductive textile thermocouples during wearing under different weather conditions is still underreported in the current literature. The influence of use and wear conditions on the durability of textile thermocouples is due to effects of moisture and low or elevated temperature. Additionally, the abrasion and mechanical deformation of conductive textile thermocouples increase the rate of degradation, which is often due to limited adhesion between the textile and conductor materials. The future scientific work should focus on the loss conductivity of textile thermocouples during aging and in situ mechanical deformation. Comprehensive scientific work is required to optimize the design, lifetime, and miniaturization of textile thermocouples. This work must include the life cycle assessment of conductive textile thermocouples to prevent hazardous waste, reduce production costs, and provide appropriate strategies for their recycling. 

## Figures and Tables

**Table 1:** Thermocouples used for temperature measurement in textiles.

Materials Used	Technique of Incorporation in Textiles	Reference	Limitations	Advantages
Cu, constantan, PES/CO fabric	Weaving	[28]	Conductive	Thermal insulation
Cu, constantan	Weaving	[27]	Stiffness	Direct application in T-shirts
Polyacrylonitrile thread, steel thread, polyamide thread, polyacrylonitrile yarn, steel fibers, graphite non-woven	Electrical conductive glue	[26]	Stiffness	Direct application in T-shirts
Stainless steel and constantan	Embroidery	[9]	Stiffness	Direct application into mattress
Poly(3,4-ethylendioxythiophene): poly(4 styrenesulfonate), polyaniline	Screen printing	[23]	Conductive	Sensitivity of 10 µV/K
Cu, constantan	Attached on polyester foam	[29]	Stiffness	Adapts to any textile structure
Cu-Ni wires, constantan wires	Soldering	[25]	Stiffness	Direct application in fire fighter gloves

**Table 2:** Temperature measurement techniques in textiles.

Measurement Technique	Set Up	Reference	Advantages
PTC	Cr/Au metal electrode structure	[30]	Sensitivity of 1.175 Ω/°C
PTC	Au/Cu contacts as flexible thermistor on the Kapton foil	[32]	Elastic conductive paste enhance flexibility
PTC	Polyurethane-coated copper filaments used as temperature-sensing textile	[33]	Construction of circuits
PTC	Activated carbon fiber cloth used as heating textile clamped between stainless steel electrodes	[31]	Measure temperature up to 200 °C
NTC	Two brass blocks placed on conductive fabric	[35]	Fast measurement
NTC	Multiwalled carbon nanotube coated poly(methylmethacrylate) yarn placed on metal clamps	[34]	Measure temperature up to 850 °C
NTC	Sensor yarns connected to microcontroller	[36]	Fast sensor integration
RTD	Conductive metal thread connected to sensor unit	[37]	Detect temperature, relative humidity
RTD	Copper electrodes	[38]	Nanocomposites enhance flexibility
RTD	Single platinum metal sensor stripes woven into textile	[39]	Fabrication of 120 sensors on one substrate
RTD	Platinum wire embedded into polyester fabric as sensing element	[40]	Knitting method similar to standard industrial process
RTD	Graphene woven fabrics and polydimethylsiloxane used as temperature sensing unit	[41]	Detect temperature, humidity
FBG	Sensors embedded into fabric by cobalt naphthenate and methyl ethyl ketone peroxide resin mixtures	[10]	Temperature sensitivity 150 pm/°C
FBG	Cladding-etched fibers used as temperature-sensing substrates	[43]	Glycerin/water compensate Bragg wavelength shift
FBG	Flexible optic fiber sensor embedded in sock reflecting the infrared light to the infrared detector	[42]	Measure temperature, pressure, joint angles
FBG	Sensors woven into a carbon fiber fabric	[44]	12 sensors measure temperature on surface and through the thickness

## References

[1] Bonauto D., Anderson R., Rauser E., Burke B. (2007). Occupational heat illness in Washington State, 1995–2005. Am. J. Ind. Med..

[2] Donoghue A.M. (2004). Heat illness in the U.S. mining industry. Am. J. Ind. Med..

[3] Howe A.S., Boden B.P. (2007). Heat-Related Illness in Athletes. Am. J. Sports Med..

[4] Xu X., Karis A.J., Buller M.J., Santee W.R. (2013). Relationship between core temperature, skin temperature, and heat flux during exercise in heat. Graefe’s Arch. Clin. Exp. Ophthalmol..

[5] Niedermann R., Psikuta A., Rossi R.M. (2014). Heat flux measurements for use in physiological and clothing research. Int. J. Biometeorol..

[6] Leonov V. (2013). Thermoelectric Energy Harvesting of Human Body Heat for Wearable Sensors. IEEE Sensors J..

[7] Morozumi Y., Akaki K., Tanabe N. (2012). Heat and moisture transfer in gaps between sweating imitation skin and nonwoven cloth: effect of gap space and alignment of skin and clothing on the moisture transfer. Heat Mass Transf..

[8] Gibson P., Charmchi M. (1997). Coupled Heat and Mass Transfer Though Hygroscopic Porous Materials—Application to Clothing Layers. Sen’I Gakkaishi.

[9] Dias T., Dias T. (2015). Electronic Textiles: Smart Fabrics and Wearable Technology.

[10] Li H., Yang H., Li E., Liu Z., Wei K. (2012). Wearable sensors in intelligent clothing for measuring human body temperature based on optical fiber Bragg grating. Opt. Express.

[11] Michalski L., Eckersdorf K., Kucharski J., McGhee J. (2001). Temperature Measurement.

[12] Lee H.S. (2010). Thermal Design: Heat Sinks, Thermoelectrics, Heat Pipes, Compact Heat Exchangers, and Solar Cells.

[13] Nicholas J.V., White D.R. (2001). Traceable Temperatures.

[14] Göpel W., Hesse J., Zemel J.N. (1990). Thermal Sensors.

[15] Senturia D.S., Wedlock D.B. (1975). Electronic Circuits and Applications.

[16] McCall C.I. US 3284247 1966, 1–3. Espacene. https://worldwide.espacenet.com/patent/search/family/023136070/publication/US3284247A?q=pn%3DUS3284247A.

[17] Daily N.J., Poteet F.R., Rahn W.M., Welch D.L. US 6550963B2 2003. Espacenet. https://worldwide.espacenet.com/patent/search/family/025288530/publication/US2003016730A1?q=US%206550963B2.

[18] Usher J.D., Blaze J.E., Phillippi M.R. US 5071258 1991. Espacenet. https://worldwide.espacenet.com/patent/search/family/024605372/publication/US5071258A?q=US%205071258%20.

[19] Smith E.T., Cooper L.C. US 20050257822A1 2005. Espacenet. https://worldwide.espacenet.com/patent/search/family/034970526/publication/US2005257822A1?q=US2005257822.

[20] Liu H., Sun W., Xu S. (2012). An Extremely Simple Thermocouple Made of a Single Layer of Metal. Adv. Mater..

[21] Sun W., Liu H., Gong W., Peng L.-M., Xu S.-Y. (2011). Unexpected size effect in the thermopower of thin-film stripes. J. Appl. Phys..

[22] Basko D., Basko D. (2011). A Photothermoelectric Effect in Graphene. Appl. Phys..

[23] Seeberg T.M., Røyset A., Jahren S., Strisland F. Printed organic conductive polymers thermocouples in textile and smart clothing applications. Proceedings of the 2011 Annual International Conference of the IEEE Engineering in Medicine and Biology Society.

[24] Depla D., Segers S., Leroy W., Van Hove T., Van Parys M. (2011). Smart textiles: an explorative study of the use of magnetron sputter deposition. Text. Res. J..

[25] Mrugala D., Ziegler F., Kostelnik J., Lang W. (2012). Temperature Sensor Measurement System for Firefighter Gloves. Procedia Eng..

[26] Ziegler S., Frydrysiak M. (2009). Initial research into the structure and working conditions of textile thermocouples. Fibres Text. East. Eur..

[27] Takatera M., Uchiyama E., Zhu C., Kim K., Ishizawa H. (2017). Effect of air gap on apparent temperature of body wearing various sizes of T-shirt. IOP Conf. Series: Mater. Sci. Eng..

[28] Gidik H., Bedek G., Dupont D., Codau C. (2015). Impact of the textile substrate on the heat transfer of a textile heat flux sensor. Sensors Actuators A: Phys..

[29] Zhu C., Takatera M. (2014). A new thermocouple technique for the precise measurement of in-plane capillary water flow within fabrics. Text. Res. J..

[30] Ataman C., Kinkeldei T., Quintero A.V., Molina-Lopez F., Courbat J., Cherenack K., Briand D., Troster G., De Rooij N. (2011). Humidity and Temperature Sensors on Plastic Foil for Textile Integration. Procedia Eng..

[31] Johnsen D.L., Rood M.J. (2012). Temperature Control during Regeneration of Activated Carbon Fiber Cloth with Resistance-Feedback. Environ. Sci. Technol..

[32] Bielska S., Sibiński M., Lukasik A. (2009). Polymer temperature sensor for textronic applications. Mater. Sci. Eng. B.

[33] Roh J.S., Kim S. (2016). All-fabric intelligent temperature regulation system for smart clothing applications. J. Intell. Mater. Syst. Struct..

[34] Sibiński M., Jakubowska M., Sloma M. (2010). Flexible Temperature Sensors on Fibers. Sensors.

[35] Pola T., Vanhala J. (2011). Resistance Measurements in Conductive Fabrics. Adv. Mater. Res..

[36] Hughes-Riley T., Lugoda P., Dias T., Trabi C.L., Morris R.H. (2017). A Study of Thermistor Performance within a Textile Structure. Sensors.

[37] Kinkeldei T., Zysset C., Cherenack K., Troster G. (2011). A textile integrated sensor system for monitoring humidity and temperature. Proceedings of the 2011 16th International Solid-State Sensors, Actuators and Microsystems Conference.

[38] Blasdel N.J., Wujcik E.K., Carletta J.E., Lee K.S., Monty C.N. (2015). Fabric nanocomposite resistance temperature detector. IEEE Sens. J..

[39] Kinkeldei T., Zysset C., Cherenack K., Troester G. Development and evaluation of temperature sensors for textile integration. Proceedings of the 2009 IEEE Sensors.

[40] Husain M.D., Kennon R., Dias T. (2014). Design and fabrication of Temperature Sensing Fabric. J. Ind. Text..

[41] Zhao X., Long Y., Yang T., Li J., Zhu H. (2017). Simultaneous High Sensitivity Sensing of Temperature and Humidity with Graphene Woven Fabrics. ACS Appl. Mater. Interfaces.

[42] Najafi B., Mohseni H., Grewal G.S., Talal T.K., Menzies R.A., Armstrong D.G. (2017). An Optical-Fiber-Based Smart Textile (Smart Socks) to Manage Biomechanical Risk Factors Associated With Diabetic Foot Amputation. J. Diabetes Sci. Technol..

[43] Kim K.T., Kim I.S., Lee C.-H., Lee J. (2012). A Temperature-Insensitive Cladding-Etched Fiber Bragg Grating Using a Liquid Mixture with a Negative Thermo-Optic Coefficient. Sensors.

[44] Jenkins R.B., Joyce P., Mechtel D. (2017). Localized Temperature Variations in Laser-Irradiated Composites with Embedded Fiber Bragg Grating Sensors. Sensors.

[45] Moraleda A.T., García C.V., Zaballa J.Z., Arrue J. (2013). A Temperature Sensor Based on a Polymer Optical Fiber Macro-Bend. Sensors.

[46] Korganbayev S., Min R., Jelbuldina M., Hu X., Caucheteur C., Bang O., Ortega B., Marques C., Tosi D., Rui M. (2018). Thermal Profile Detection Through High-Sensitivity Fiber Optic Chirped Bragg Grating on Microstructured PMMA Fiber. J. Light. Technol..

[47] Min R., Ortega B., Marques C. (2019). Latest Achievements in Polymer Optical Fiber Gratings: Fabrication and Applications. Photonics.

[48] Xiang Z., Wan L., Gong Z., Zhou Z., Ma Z., Ouyang X., He Z., Chan C.C. (2019). Multifunctional Textile Platform for Fiber Optic Wearable Temperature-Monitoring Application. Micromachines.

[49] Post E.R., Orth M., Russo P.R., Gershenfeld N. (2000). E-broidery: Design and fabrication of textile-based computing. IBM Syst. J..

[50] Nilsson E., Lund A., Jonasson C., Johansson C., Hagström B. (2013). Poling and characterization of piezoelectric polymer fibers for use in textile sensors. Sensors Actuators A: Phys..

[51] Ramachandran T., Vigneswaran C. (2009). Design and Development of Copper Core Conductive Fabrics for Smart Textiles. J. Ind. Text..

[52] Matsuhisa N., Kaltenbrunner M., Yokota T., Jinno H., Kuribara K., Sekitani T., Someya T. (2015). Printable elastic conductors with a high conductivity for electronic textile applications. Nat. Commun..

[53] Satharasinghe A.S., Jayasundara H., Vitarana R.K. US 20160194792A1 2016. Espacenet. https://worldwide.espacenet.com/patent/search/family/053765507/publication/US2016194792A1?q=US20160194792A1%20.

[54] Li L., Fan T., Hu R., Liu Y., Lu M. (2017). Surface micro-dissolution process for embedding carbon nanotubes on cotton fabric as a conductive textile. Cellulose.

[55] Tao X., Mei-Yi Leung Leung S., Chun-Wah Yuen M., Kwok W.-Y., Ho H.-L. US 20060148351A1 2006, 1. Espacenet. https://worldwide.espacenet.com/patent/search/family/036641162/publication/US2006148351A1?q=US20060148351A1%20.

[56] Amelio W.J., Lemon G., Markovich V., Panasik T., Sambucetti C., Trevitt D. US 4,448,804 1984. Espacenet. https://worldwide.espacenet.com/patent/search/family/024156146/publication/US4448804A?q=US4448804%20.

[57] Wills K.A., Krzyzak K., Bush J., Ashayer-Soltani R., Graves J.E., Hunt C., Cobley A.J. (2017). Additive process for patterned metallized conductive tracks on cotton with applications in smart textiles. J. Text. Inst..

[58] Post E.R., Orth M. (1997). Smart fabric, or ‘wearable clothing’. Digest of Papers.

[59] Ashayer-Soltani R., Hunt C.P. WO2014128505A1 SR.pdf 2014. Espacenet. https://worldwide.espacenet.com/patent/search/family/048092026/publication/WO2014128505A1?q=WO2014128505A1.

[60] Root W., Aguiló-Aguayo N., Pham T., Bechtold T. (2018). Conductive layers through electroless deposition of copper on woven cellulose lyocell fabrics. Surf. Coatings Technol..

[61] Schlesinger M., Paunovic M. (2010). Modern electroplating.

[62] Ali A., Baheti V., Militky J., Khan Z., Tunakova V., Naeem S. (2018). Copper coated multifunctional cotton fabrics. J. Ind. Text..

[63] Li L., Au W.M., Ding F., Hua T., Wong K.S. (2014). Wearable electronic design: Electrothermal properties of conductive knitted fabrics. Text. Res. J..

[64] Krucinska I., Surma B., Chrzanowski M. (2010). Study on Sensing Properties of Electro-spun PEO/MWNT Non-woven Fabric. Res. J. Text. Appar..

[65] Ferreira A., Ferreira F., Paiva M.C. (2012). Textile Sensor Applications with Composite Monofilaments of Polymer/Carbon Nanotubes. Adv. Sci. Technol..

[66] Daoud W.A., Xin J.H., Szeto Y.S. (2005). Polyethylenedioxythiophene coatings for humidity, temperature and strain sensing polyamide fibers. Sensors Actuators B: Chem..

[67] Cheng Y., Zhang H., Wang R., Wang X., Zhai H., Wang T., Jin Q., Sun J. (2016). Highly Stretchable and Conductive Copper Nanowire Based Fibers with Hierarchical Structure for Wearable Heaters. ACS Appl. Mater. Interfaces.

[68] Dolez P.I., Vu-Khanh T. (2009). Recent Developments and Needs in Materials Used for Personal Protective Equipment and Their Testing. Int. J. Occup. Saf. Ergon..

[69] Rossi R.M., Bolli W., Stämpfli R. (2008). Performance of Firefighters’ Protective Clothing After Heat Exposure. Int. J. Occup. Saf. Ergon..

[70] Stoppa M., Chiolerio A. (2014). Wearable Electronics and Smart Textiles: A Critical Review. Sensors.

[71] Jiang X., Tessier D., Zhang Z. (2002). Biostability of electrically conductive polyester fabrics: Anin vitro study. J. Biomed. Mater. Res..

[72] Wearable Technology 2015-2025: Technologies, Markets, Forecast E-Textiles, Wearable Electronics, Medicals Diagnostics/Telemedicine, Smart Glasses, Smart Wristbands and More. By Dr Peter Harrop, Mr James Hayward, Raghu Das and Glyn Holland. IDTechEx. https://www.idtechex.com/ja/research-report/wearable-technology-2015-2025-technologies-markets-forecasts/427?setlang=ja.

[73] Google. https://ec.europa.eu/digital-single-market/en/news/feedback-stakeholders-smart-wearables-reflection-and-orientation-paper.

[74] Köhler A.R. (2013). Challenges for eco-design of emerging technologies: The case of electronic textiles. Mater. Des..

[75] Rebitzer G., Ekvall T., Frischknecht R., Hunkeler D., Norris G., Rydberg T., Suh S., Weidema B.P., Pennington D.W. (2004). Life cycle assessment Part 1: Framework, goal and scope definition, inventory analysis, and applications. Environ. Int..

[76] Mantecca P., Kasemets K., Deokar A., Perelshtein I., Gedanken A., Bahk Y.K., Kianfar B., Wang J. (2017). Airborne Nanoparticle Release and Toxicological Risk from Metal-Oxide-Coated Textiles: Toward a Multiscale Safe-by-Design Approach. Environ. Sci. Technol..

[77] Köhler A.R., Hilty L.M., Bakker C. (2011). Prospective Impacts of Electronic Textiles on Recycling and Disposal. J. Ind. Ecol..

[78] Kutz M. (2007). Environmentally Conscious Manufacturing.

[79] Jang J., Hyun B.G., Ji S., Cho E., An B.W., Cheong W.H., Park J.-U. (2017). Rapid production of large-area, transparent and stretchable electrodes using metal nanofibers as wirelessly operated wearable heaters. NPG Asia Mater..

[80] Zhou R., Li P., Fan Z., Du D., Ouyang J. (2017). Stretchable heaters with composites of an intrinsically conductive polymer, reduced graphene oxide and an elastomer for wearable thermotherapy. J. Mater. Chem. C.

[81] Ji S., He W., Wang K., Ran Y., Ye C. (2014). Thermal Response of Transparent Silver Nanowire/PEDOT:PSS Film Heaters. Small.

[82] Wang R., Xu Z., Zhuang J., Liu Z., Peng L., Li Z., Liu Y., Gao W., Gao C. (2017). Highly Stretchable Graphene Fibers with Ultrafast Electrothermal Response for Low-Voltage Wearable Heaters. Adv. Electron. Mater..

[83] Li Y., Zhang Z., Li X., Zhang J., Lou H., Shi X., Cheng X., Peng H. (2017). A smart, stretchable resistive heater textile. J. Mater. Chem. C.

[84] Jo H.S., An S., Lee J.-G., Park H.G., Al-Deyab S.S., Yarin A.L., Yoon S.S. (2017). Highly flexible, stretchable, patternable, transparent copper fiber heater on a complex 3D surface. NPG Asia Mater..

[85] Kim C.-L., Lee J.-J., Oh Y.-J., Kim D.-E. (2017). Smart wearable heaters with high durability, flexibility, water-repellent and shape memory characteristics. Compos. Sci. Technol..

[86] Yeon C., Kim G., Lim J.W., Yun S.J. (2017). Highly conductive PEDOT:PSS treated by sodium dodecyl sulfate for stretchable fabric heaters. RSC Adv..

[87] Choi S., Park J., Hyun W., Kim J., Kim J., Lee Y.B., Song C., Hwang H.J., Kim J.H., Hyeon T. (2015). Stretchable Heater Using Ligand-Exchanged Silver Nanowire Nanocomposite for Wearable Articular Thermotherapy. ACS Nano.

[88] Jang N.-S., Kim K.-H., Ha S.-H., Jung S.-H., Lee H.M., Kim J.-M. (2017). Simple Approach to High-Performance Stretchable Heaters Based on Kirigami Patterning of Conductive Paper for Wearable Thermotherapy Applications. ACS Appl. Mater. Interfaces.

[89] Li P., Ma J., Xu H., Xue X., Liu Y. (2016). Highly stable copper wire/alumina/polyimide composite films for stretchable and transparent heaters. J. Mater. Chem. C.

[90] An B.W., Gwak E.J., Kim K., Kim Y.C., Jang J., Kim J.Y., Park J.U. (2016). Stretchable, Transparent Electrodes as Wearable Heaters Using Nanotrough Networks of Metallic Glasses with Superior Mechanical Properties and Thermal Stability. Nano Lett..

[91] Hong S., Lee H., Lee J., Kwon J., Han S., Suh Y.D., Cho H., Shin J., Yeo J., Ko S.H. (2015). Highly Stretchable and Transparent Metal Nanowire Heater for Wearable Electronics Applications. Adv. Mater..

[92] Cherenack K., Zysset C., Kinkeldei T., Münzenrieder N., Tröster G. (2010). Woven Electronic Fibers with Sensing and Display Functions for Smart Textiles. Adv. Mater..

[93] Du Y., Xu J., Wang Y., Lin T. (2017). Thermoelectric properties of graphite-PEDOT:PSS coated flexible polyester fabrics. J. Mater. Sci. Mater. Electron..

[94] Tien N.T., Jeon S., Il Kim D., Trung T.Q., Jang M., Hwang B.U., Byun K.E., Bae J., Lee E., Tok J.B.H. (2014). A Flexible Bimodal Sensor Array For Simultaneous Sensing of Pressure and Temperature. Adv. Mater..

[95] Neves S., Campos J., Mayor T.S. (2015). On the determination of parameters required for numerical studies of heat and mass transfer through textiles – Methodologies and experimental procedures. Int. J. Heat Mass Transf..

[96] Huang J., Chen Y. (2010). Effects of Air Temperature, Relative Humidity, and Wind Speed on Water Vapor Transmission Rate of Fabrics. Text. Res. J..

[97] Yao S., Zhu Y. (2015). Nanomaterial-Enabled Stretchable Conductors: Strategies, Materials and Devices. Adv. Mater..

[98] Luprano J. (2008). Bio-Sensing Textile for Medical Monitoring Applications. Adv. Sci. Technol..

[99] Kim S.J., We J.H., Cho B.J. (2014). A wearable thermoelectric generator fabricated on a glass fabric. Energy Environ. Sci..

[100] Jeon J., Lee H.B.R., Bao Z. (2013). Flexible Wireless Temperature Sensors Based on Ni Microparticle-Filled Binary Polymer Composites. Adv. Mater..

[101] Ueno S., Sawada S. (2012). Correction of the evaporative resistance of clothing by the temperature of skin fabric on a sweating and walking thermal manikin. Text. Res. J..

[102] Wang D., Zhang Y., Lu X., Ma Z., Xie C., Zheng Z. (2018). Chemical formation of soft metal electrodes for flexible and wearable electronics. Chem. Soc. Rev..

